# *Ruminococcus gnavus* bacteraemia showing morphological diversity on Gram staining: a case report and literature review

**DOI:** 10.1099/acmi.0.000442

**Published:** 2023-06-22

**Authors:** Tatsuya Hioki, Kensuke Kataoka, Yoshikazu Mutoh

**Affiliations:** ^1^​ Department of Clinical Laboratory, Tosei General Hospital, 160 Nishioiwake-cho, Seto, Aichi, 489-8642, Japan; ^2^​ Department of Respiratory Medicine and Allergy, Tosei General Hospital, 160 Nishioiwake-cho, Seto, Aichi, 489-8642, Japan; ^3^​ Department of Infectious Diseases, Tosei General Hospital, 160 Nishioiwake-cho, Seto, Aichi, 489-8642, Japan

**Keywords:** bacteraemia, Gram staining, matrix-assisted laser desorption/ionization time-of-flight mass spectrometry, *Ruminococcus gnavus*

## Abstract

*
Ruminococcus gnavus *, a Gram-positive anaerobic coccus, is a common constituent of the human gut microbiota but rarely causes any disease in humans. Herein, we report a case of *R. gnavus* bacteraemia in an immunocompromised 73-year-old man with sigmoid colon perforation. *R. gnavus* is usually reported as Gram-positive diplococci or short chains on Gram staining; however, in our patient, a blood isolate showed Gram-positive cocci in long chains, and organisms from an anaerobic subculture showed morphological diversity. This case provides insight into the morphological diversity of *R. gnavus *, which might help with the recognition of these bacteria in the preliminary identification stage on Gram staining.

## Introduction

*Ruminococcus gnavus *, an anaerobic Gram-positive coccus, is a common constituent of the human gut microbiota but rarely causes any disease. We found only 11 case reports of *R. gnavus* infection [1–10] in a literature search, including six cases of *R. gnavus* bacteraemia [1–5]. The morphology of *R. gnavus *, including in blood cultures, is usually described as diplococci or short chains; however, few reports have focused on the Gram staining morphology. In this report, we describe a case of bacteraemia caused by *R. gnavus* that showed morphological diversity on Gram staining in an immunocompromised patient with sigmoid colon perforation.

## Case presentation

A 73-year-old man undergoing steroid and immunosuppressive therapy for interstitial pneumonia was admitted to our hospital complaining of difficulty breathing. He had experienced repeated exacerbations of interstitial pneumonia and had been treated with prednisolone and tacrolimus. He had received oral sulfamethoxazole-trimethoprim (ST) for prophylaxis of *Pneumocystis* pneumonia. Additionally, he had been treated with oral amphotericin B for oral candidiasis 2 months previously. His medical history included pneumatosis intestinalis and diabetes mellitus secondary to steroid therapy. On admission, blood tests revealed a normal C-reactive protein level (0.24 mg dl^−1^) and an elevated white blood cell count (12.6×10^9^ cells l^−1^) with 94.4 % neutrophils. The patient was suspected of having a chronic exacerbation of interstitial pneumonia and was hospitalized. During hospitalization, he received an antihyperglycaemic regimen and underwent further investigations such as lung function tests and pulmonary rehabilitation. Immunosuppressive therapy with prednisolone and tacrolimus had been continued. His respiratory symptoms improved on the 40th day in hospital, but he suddenly developed acute abdominal pain.

A computed tomography scan of the abdomen revealed free air and some faeces in the abdominal cavity, confirming intestinal perforation. Two sets of blood cultures consisting of aerobic and anaerobic bottles (Becton Dickinson Plus Aerobic/F, Plus Anaerobic/F; Becton Dickinson and Company) were performed using the BD BACTEC FX system (Becton Dickinson and Company), and empiric antibiotic therapy with meropenem was initiated. Emergency laparotomy was performed, which revealed a perforation in the sigmoid colon with leakage of stools and food residue into the abdominal cavity. Therefore, sigmoid colostomy as well as thorough drainage and intra-abdominal irrigation were performed. The patient was admitted to the intensive care unit for postoperative management, where he was treated with fluid therapy, vasopressors and mechanical ventilation in addition to antibiotic treatment with meropenem. As his general condition and hypotension did not improve, polymyxin B-immobilized fibre column-direct haemoperfusion therapy was performed, which resulted in haemodynamic stabilization.

**Fig. 1.**
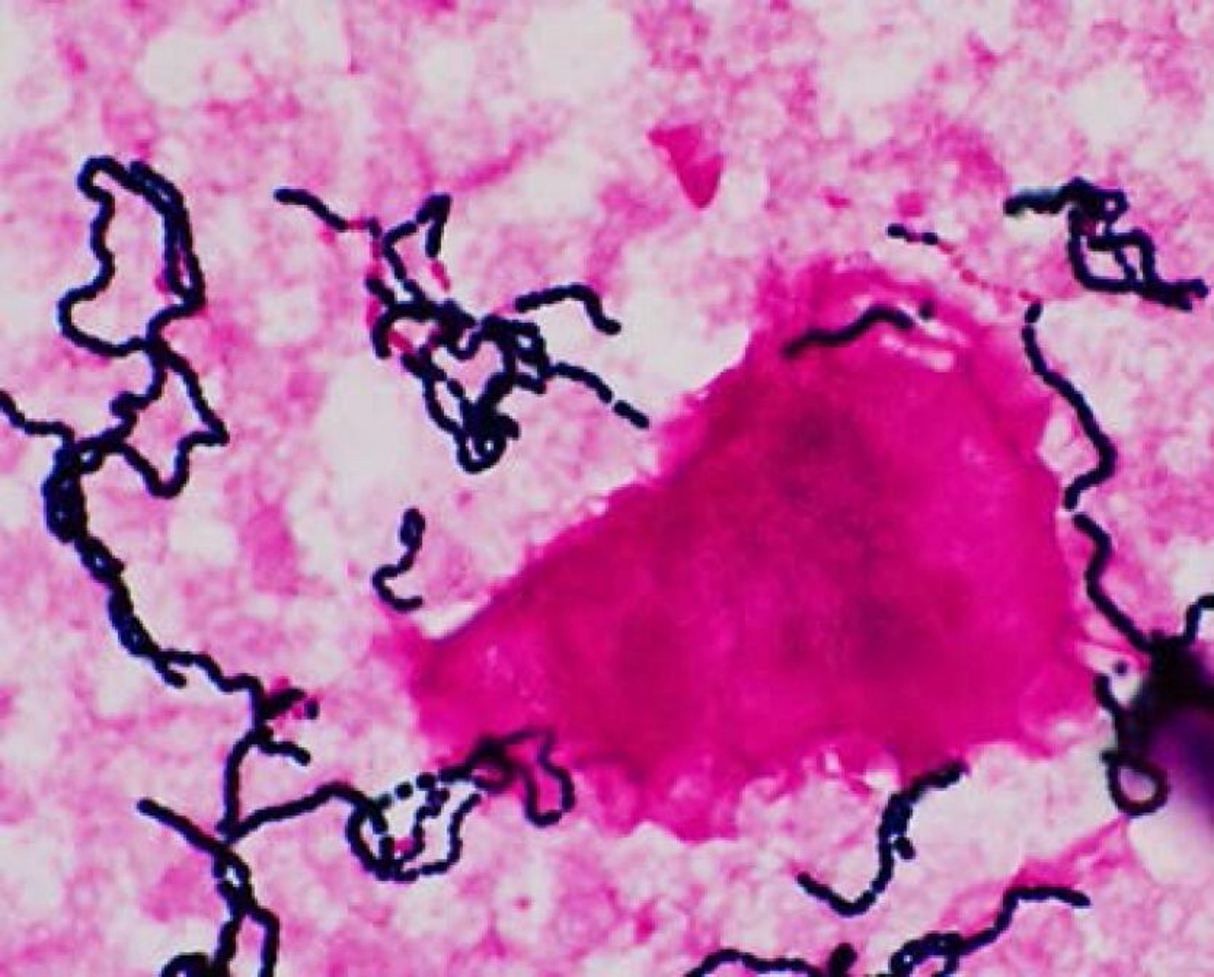
Gram staining of blood culture showing Gram-positive cocci in long chains and some decolorized Gram-negative bacteria (×1000 magnification).

Of the blood cultures, one anaerobic bottle was positive after 30.5 h of incubation at 35 °C; the others remained negative even after 6 days of incubation. Gram staining revealed Gram-positive cocci in long chains with some decolorized segments (Fig. 1). Subcultures were performed on 5 % Sheep Blood Agar (Becton Dickinson and Company) in 5 % CO_2_ at 37 °C and under anaerobic conditions at 35 °C. Several translucent small bacterial colonies were observed in the anaerobic culture after 24 h of incubation. These colonies were the same as for the Gram-positive bacteria seen on the blood culture; however, unlike the blood culture, they showed morphological variety characterized by large diplococci, short chains, and swollen and elongated forms.

A biochemical test (Rapid ID32A V3.3; bioMérieux) was performed. The bacteria were positive for α-galactosidase, β-galactosidase, α-glucosidase, β-glucosidase and raffinose fermentation, and weakly positive for α-fucosidase. The biochemical test showed the presence of *Clostridium* species with a low identification probability; therefore, additional testing was performed using matrix-assisted laser desorption/ionization time-of-flight MS (MALDI-TOF MS) (MALDI Biotyper software version 4.1.90/BDAL7854, version 8; Bruker Daltonik). The cell smear method showed *R. gnavus* with an insufficient score (1.73) for species confirmation; however, a repeat test performed using the formic acid/ethanol tube extraction method provided a sufficient score (2.24) for species identification of *R. gnavus *.

Furthermore, antimicrobial susceptibility testing was performed using the Kyokuto Opt Panel MP OP1 (Kyokuto Pharmaceutical Industrial) microdilution method, and the MIC was determined for benzylpenicillin (<0.06 μg ml^−1^), cefotaxime (<0.5 μg ml^−1^), imipenem (<0.12 μg ml^−1^), meropenem (<0.12 μg ml^−1^), clindamycin (<0.12 μg ml^−1^), erythromycin (>2 μg ml^−1^) and levofloxacin (>8 μg ml^−1^). Additionally, the MICs of vancomycin (0.38 μg ml^−1^) and metronidazole (0.032 μg ml^−1^) were measured using the Etest on the Anaero Columbia Agar with Rabbit Sheep Blood (Becton Dickinson and Company) supplemented with vitamin K1 and hemin.

In addition, peritoneal fluid collected during surgery was cultured. Bacterial growth of *Escherichia coli *, *Enterococcus avium* and *Enterococcus faecalis* was observed, but no anaerobic bacteria were isolated. For each isolate, antimicrobial susceptibility testing was performed using the MicroScan WalkAway 96 (Beckman Coulter), based on the microdilution method and the Clinical and Laboratory Standards Institute criteria. The *Escherichia coli* strain was an extended-spectrum beta-lactamase (ESBL) producer; therefore, antibiotic treatment with meropenem was continued for 23 days. Subsequently, repeat blood cultures were negative.

**Fig. 2.**
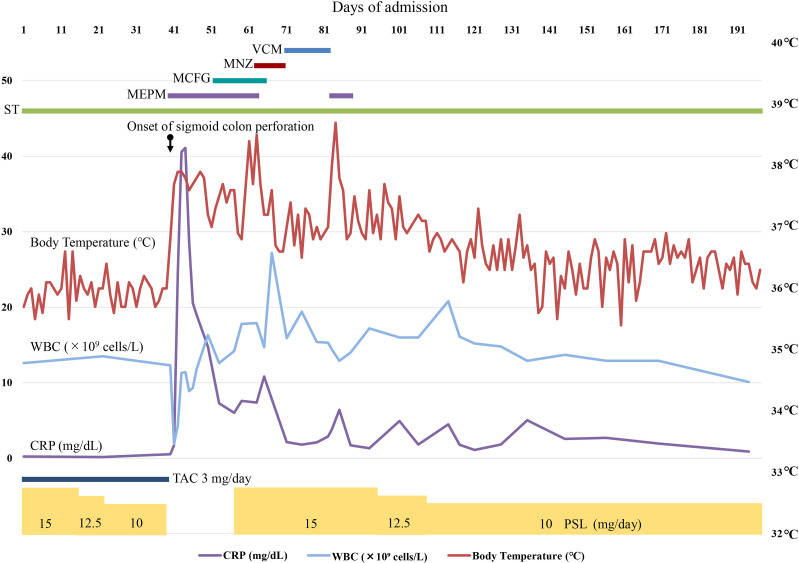
Timeline of the patient’s clinical course and antibiotic treatment. The *y*-axis on the left shows the white blood cell (WBC) count and the C-reactive protein (CRP) level. The *y*-axis on the right shows body temperature.

Seven days after surgery, the inflammatory response gradually improved; however, a surgical site infection (SSI) developed. As in the peritoneal fluid, ESBL-producing *Escherichia coli* and *Enterococcus avium* were detected in the wound. The SSI resolved after wound cleaning and meropenem treatment. Nine days after surgery, a blood test showed an elevated plasma β-d-glucan level. *Candida* peritonitis was suspected, and intravenous micafungin was initiated. Three weeks later, the patient developed watery diarrhoea caused by *Clostridioides difficile* infection (CDI), which was diagnosed by detecting the toxigenic *C. difficile* strain using an Xpert *C. difficile* assay (Cepheid). Oral metronidazole was administered first but was replaced with oral vancomycin because CDI was refractory to metronidazole. Six weeks after surgery, fever and an elevated C-reactive protein level were detected, which were suspected to be caused by SSI. These improved rapidly following a further 6 day course of meropenem. These problems made the treatment more difficult; however, the signs of infection improved, and the patient was discharged after 5 months. The clinical course of the patient is illustrated in Fig. 2.

## Discussion

We encountered a case of *R. gnavus* bacteraemia in which the cultured organisms showed morphological diversity on Gram staining. A literature search found only 11 previously reported cases of *R. gnavus* infection, including only six cases of bacteraemia [1–10] (Table 1). All the reported cases of bacteraemia originated from the digestive tract [1–5]. Four cases of infection involved patients with joint prostheses or septic arthritis without an implant [6–9]. Of these four cases, two were suspected to be associated with digestive disease [8, 9]; in the remaining two cases, it was unclear whether the bacteria were introduced by the prostheses or were due to haematogenous dissemination [6, 7]. There was also one case report of bilateral tubo-ovarian abscesses caused by *R. gnavus* infection [10]. Several studies have reported that the relative abundance of *R. gnavus* is significantly higher in patients with inflammatory bowel disease than in healthy individuals, and this increased abundance of *R. gnavus* was transient, which often corresponded to increased disease activity [11].

**Table 1.** Previous case reports of *Ruminococcus gnavus* infection

Reference	Year	Age (years), sex	Case report	Sample	Identification method	Gram staining of blood culture
[1]	2013	67 M	Diverticulitis perforation	Blood	MALDI-TOF MS, 16S rRNA gene sequencing	Large Gram-positive diplococci
[1]	2013	90 M	Sigmoid colon diverticulosis without perforation	Blood	MALDI-TOF MS, 16S rRNA gene sequencing	Gram-positive diplococci
[2]	2017	82 F	Gall bladder perforation and cholecystitis	Blood	MALDI-TOF MS, 16S rRNA gene sequencing	Gram-positive diplococci and chains
[3]	2018	77 M	Sigmoid colon perforation	Blood	MALDI-TOF MS, 16S rRNA gene sequencing	Gram-positive diplococci and some small chains
[4]	2018	66 F	Small bowel strangulated hernia and perforation	Blood	MALDI-TOF MS, 16S rRNA gene sequencing	Large Gram-positive diplococci
[5]	2019	76 F	Vulnerable mucosal barrier (history of minor gastrointestinal bleeding)	Blood	MALDI-TOF MS	Gram-positive cocci in chains
[6]	2017	72 F	Prosthetic joint infection	Granuloma, pseudotumour samples, prosthetic components	MALDI-TOF MS	—
[7]	2017	93 F	Prosthetic joint infection	Prosthetic material	MALDI-TOF MS	—
[8]	2015	62 M	Prosthetic joint infection, ulcerative colitis	Bone biopsy sample	MALDI-TOF MS, 16S rRNA gene sequencing	—
[9]	2014	47 M	Septic arthritis without an implant (suspected gastrointestinal fistula)	Joint fluid	MALDI-TOF MS, 16S rRNA gene sequencing	—
[10]	2021	27 F	Bilateral tubo-ovarian abscesses	Drainage of ovarian abscesses	MALDI-TOF MS	—

In addition, it has been reported that mucin destruction and glucorhamnan production by *R. gnavus* affect human inflammatory responses [12]. However, it is unclear whether *R. gnavus* is directly involved in exacerbating this condition. Similar to our case report, these reports also reveal that digestive disease is an important source of *R. gnavus* infection. In addition, it has been previously reported that an immunocompromised state is a risk factor for *R. gnavus* infection [3, 5], which is consistent with our patient having received steroid and immunosuppressive therapy. Further studies and accumulation of case reports are needed to better understand the pathogenesis of bacteraemia caused by *R. gnavus *.

MALDI-TOF MS has recently been suggested as a useful tool for detecting *R. gnavus* [3]. It is sometimes possible to identify *R. gnavus* using MALDI-TOF MS; however, the accuracy of identification varies according to the MALDI-TOF MS system and database version used [1, 4].

The morphology of *R. gnavus *, including in blood cultures, has been reported as diplococci or short chains, and the organisms are generally described as slightly elongated with tapered ends. In contrast to the typical staining appearance, the blood culture from our patient showed Gram-positive cocci in long chains, while the colonies grown on the anaerobic subculture were morphologically diverse. These growth characteristics were similar to those of nutritionally deficient streptococci (NDS). NDS appear as cocci in short chains if cultured with appropriate nutrients but develop more rod-like, filamentous, bulging and aberrant morphologies under nutrient-limited conditions [13]. Based on this, we hypothesize that the morphological changes seen in the *R. gnavus* anaerobic subculture isolated from our patient were induced by nutritional conditions and the properties of the medium.

Additionally, the differences in blood culture bottles may affect bacterial morphology. However, with Gram-negative bacteria, exposure to antibiotic agents, especially beta-lactam antibiotics, is known to have various effects on the cell wall and can induce morphological changes [14]. The administration of antibiotics such as ST and amphotericin B possibly induced morphological changes in this patient. It is difficult to identify the factors that determine morphology because there are many sources of variation [13]. The staining characteristics of some decolorized Gram-negative bacteria are consistent with a report by Kim *et al.* [2], in which they described *R. gnavus* displaying a mixture of Gram-positive diplococci and chain forms and Gram-negative decolorized cocci. Bacterial morphologies and staining characteristics are fundamental for the identification of bacteria.

Knowledge about the antimicrobial susceptibility of *R. gnavus* is limited. Most previously reported strains were susceptible to beta-lactams [1–9], metronidazole [1–5, 7–9] and vancomycin [1–4, 6, 8, 9], but resistant to fluoroquinolones [1, 2, 4, 8, 9]. Consistent with previous reports, the strain in our patient showed low MICs for multiple antibiotic agents, including meropenem, and a high MIC for levofloxacin. We treated the patient with meropenem, considering the antimicrobial susceptibility testing and the ESBL-producing *Escherichia coli* isolate. The patient was successfully treated, and the follow-up blood cultures were negative. However, antibiotic agents for treating *R. gnavus* infection must be carefully selected because resistance to benzylpenicillin [1] and clindamycin [1, 3] has been reported.

In conclusion, we report here a case of *R. gnavus* bacteraemia in which an isolate cultured under anaerobic conditions showed morphological diversity. Generally, the bacterial morphology of *R. gnavus* has been reported as diplococci or short chains but can show cocci in long chains. This case provides insight into the morphological diversity of *R. gnavus *, which might help with the recognition of these bacteria in the preliminary identification stage on Gram staining.
